# In vivo X-ray microtomography locally affects stem radial growth with no immediate physiological impact

**DOI:** 10.1093/plphys/kiae285

**Published:** 2024-05-17

**Authors:** Laura Mekarni, Hervé Cochard, Marco M Lehmann, Pascal Turberg, Charlotte Grossiord

**Affiliations:** Plant Ecology Research Laboratory PERL, School of Architecture, Civil and Environmental Engineering, EPFL, CH-1015 Lausanne, Switzerland; Swiss Federal Institute for Forest, Snow and Landscape Research WSL, Community Ecology Unit, 8903 Birmensdorf, Switzerland; Université Clermont Auvergne, INRAE, PIAF, 63000 Clermont-Ferrand, France; Swiss Federal Institute for Forest, Snow and Landscape Research WSL, Community Ecology Unit, 8903 Birmensdorf, Switzerland; Plant Ecology Research Laboratory PERL, School of Architecture, Civil and Environmental Engineering, EPFL, CH-1015 Lausanne, Switzerland; Swiss Federal Institute for Forest, Snow and Landscape Research WSL, Community Ecology Unit, 8903 Birmensdorf, Switzerland; Plant Ecology Research Laboratory PERL, School of Architecture, Civil and Environmental Engineering, EPFL, CH-1015 Lausanne, Switzerland; Swiss Federal Institute for Forest, Snow and Landscape Research WSL, Community Ecology Unit, 8903 Birmensdorf, Switzerland

## Abstract

Microcomputed tomography (µCT) is a nondestructive X-ray imaging method used in plant physiology to visualize in situ plant tissues that enables assessments of embolized xylem vessels. Whereas evidence for X-ray-induced cellular damage has been reported, the impact on plant physiological processes such as carbon (C) uptake, transport, and use is unknown. Yet, these damages could be particularly relevant for studies that track embolism and C fluxes over time. We examined the physiological consequences of µCT scanning for xylem embolism over 3 mo by monitoring net photosynthesis (*A*_net_), diameter growth, chlorophyll (Chl) concentration, and foliar nonstructural carbohydrate (NSC) content in 4 deciduous tree species: hedge maple (*Acer campestre*), ash (*Fraxinus excelsior*), European hornbeam (*Carpinus betulus*), and sessile oak (*Quercus petraea*). C transport from the canopy to the roots was also assessed through ^13^C labeling. Our results show that monthly X-ray application did not impact foliar A_net_, Chl, NSC content, and C transport. Although X-ray effects did not vary between species, the most pronounced impact was observed in sessile oak, marked by stopped growth and stem deformations around the irradiated area. The absence of adverse impacts on plant physiology for all the tested treatments indicates that laboratory-based µCT systems can be used with different beam energy levels and doses without threatening the integrity of plant physiology within the range of tested parameters. However, the impacts of repetitive µCT on the stem radial growth at the irradiated zone leading to deformations in sessile oak might have lasting implications for studies tracking plant embolism in the longer-term.

## Teaser

Monthly application of X-rays reduces radial growth and deforms stems in some deciduous tree species but has little impact on other physiological functions over a growing season.

## Introduction

Computed tomography (CT) is an imaging method that has gradually revolutionized the possibilities of in situ organic tissue exploration (Piovesan et al. 2021). Initially used in the medical sphere (Howell 2016), the fields of CT application have broadened, and the evolution of technologies over the past 20 yr has made it possible to achieve much higher resolutions (down to 1 *µ*m/pixel; Cochard et al. 2015), leading to the micro-CT (µCT) appellation. Recently, µCT has gained wide interest in the field of plant hydraulics because of its ability to assess nondestructively conduit structure, xylem function, and embolism formation in living plants. However, large uncertainties remain regarding the potential injuries induced by X-ray radiations on plant physiology and, subsequently, on interpreting results from studies using this approach.

The first methodology of in vivo µCT scans on plant stems to evaluate the structure and function of the xylem network in 3 dimensions was defined by McElrone et al. (2013) and used as a reference in many follow-up studies. Herbaceous (Savi et al. 2017; Skelton et al. 2017; Montanha et al. 2019; Cardoso et al. 2020; Corso et al. 2020) and woody plants (Brodersen et al. 2018; Losso et al. 2019) have since been studied, including a range of plant organs (i.e. leaves, petioles, stems, trunks, and roots) (e.g. Pajor et al. 2013; Hochberg et al. 2015; Karahara et al. 2015; Mathers et al. 2018). The standard method for woody tissues consists of mounting the living plant in a holder on the air-bearing stage of the scanner to keep the stem as vertical as possible and minimize vibrations. The position of the target tissue to be scanned is optimized to fit with the field of view while being as close as possible to the X-ray source. After geometrical reconstruction, typical µCT measurements yield a series of transverse (i.e. cross-sectional) images composed of “voxels” (i.e. volumetric pixel elements), each with an *x*, *y*, and *z* coordinate and intensity values representing the X-ray linear absorption coefficient (Vásárhelyi et al. 2020). Using such images, we can extract tissue properties such as the size and density of xylem elements. The density contrast between water- and air-filled conduits enables calculations of the theoretical percentage loss of conductivity or conductive area (PLC or PLA, respectively) in plant stems dehydrated to different xylem pressures. PLC and PLA assessments made from µCT are in good agreement with classical hydraulic techniques (Nardini et al. 2017; Nolf et al. 2017; Gauthey et al. 2020; Schönbeck et al. 2022) and have even shown more reliable results than older techniques (i.e. Cavitron) in the case of long-vessel species (Cochard et al. 2015). This imaging method has especially attracted interest for studies investigating plant's ability to recover vessel use after embolism. Indeed, multiple studies applied drought and recovery cycles to potted plants and performed µCT scans to track the hydraulic processes underway (Brodersen et al. 2010; Choat et al. 2015; Knipfer et al. 2015, 2017, 2019; Brodersen et al. 2018). Such a follow-up over time had never been possible before the advent of this so-called nondestructive method. Still, repetitive scans raise the questions of side effects on tree physiology induced by exposure to X-rays.

Even if µCT is generally defined as a nondestructive imaging technique, several physiological responses are known to be inflicted by irradiation doses. Interestingly, X-ray was first used for its stimulative effects on plant growth (Shull and Mitchell 1933), particularly if applied in low doses up to 5 Gy (Rezk et al. 2019). In contrast, higher doses induced by repeated scans could lead to cell damage and decrease the photosynthetic activity (Matsushima et al. 2013). Indeed, the concentration of photosynthetic pigments and DNA content was found to decrease as X-ray doses increased from 0.05 to 15 Gy (Al-Enezi and Al-Khayri 2012). Moreover, Petruzzellis et al. (2018) showed that multiple µCT scans (total dose applied to the stem around 8.5 Gy) could disrupt cell membranes and affect the RNA integrity in living stem cells of herbaceous and woody species. Montanha et al. (2019) further highlighted that increasing X-ray radiation doses using variable currents, scan durations, and filters can lead to greater epidermal and mesophyll cell necrosis, cell-wall breakage, and cytoplasm collapse. Moreover, in addition to the dose, the energy distribution of the X-ray beam is known to affect the intensity of biological damage (Gianoncelli et al. 2015). For example, low-energy photons are more absorbed by soft tissues like plants than high-energy ones. Thus, they are more likely to cause cell degradation. To modify the X-ray beam's energy, one can adjust the tube voltage, and undesirable low-energy photons can be further filtered out by adding filters at the beam source, a process known as beam hardening (Mahesh 2013). Overall, our limited knowledge of µCT impacts suggests that scanning even a short portion of plant stems (as for xylem embolism assessments) could necrotize cells, thereby damaging the cambium and the radial growth locally. In the same way, cell destruction could impair the phloem and transport of nonstructural carbohydrates (NSCs), which could reduce poststress hydraulic recovery in manipulative experiments (Tomasella et al. 2020). Such adverse impacts may be regulated by adapting the X-ray doses or reducing the low-energy photons using filters. Yet, no study to date has specifically addressed how repeated µCT scans at different doses or energy levels affect the growth, photosynthetic activity, and phloem transport in plants.

The emphasis of this study was on investigating the physiological impacts of scanning for xylem embolism detection, rather than exploring the complete spectrum of X-ray exposure options available with the µCT. The same seedlings from 4 different species (hedge maple—*Acer campestre*, *ash -Fraxinus excelsior*, *European hornbeam—Carpinus betulus*, and *sessile oak—Quercus petraea*) were scanned monthly over a 3-mo growing season with different X-ray energy levels and doses (tube voltage from 40 to 80 kV, doses ranging from 8 up to 22 Gy per scan). More specifically, we tested how µCT scanning applied on a short portion of the stem impacts the net light-saturated photosynthesis (*A*_net_), the foliar chlorophyll (Chl) content, the foliar NSC content, the radial growth of the stem, and the carbon (C) transport from the canopy to the roots using isotopic pulse labeling with ^13^CO_2_. Measurements were conducted on leaves above and below the µCT-scanned zone to test the effect of local stem necrosis on the leaf physiology (*A*_net_, Chl, and NSC) on either side. We expected X-rays to damage the stem cambium and phloem, leading to reduced radial growth and C transport from the upper leaves to the roots. Consequently, foliar NSC content should decrease below the scanned area because of exhausting C resources throughout the growing season. As X-rays would only be applied locally to the stem, we further expected *A*_net_ and Chl above and below the scanned area to remain stable, therefore not offsetting the decline in C resources. We further hypothesized that despite lower doses, low-energy X-rays would have stronger impacts on plant physiology (e.g. stronger growth reduction, lowered C transport, and NSC depletion) than high-energy X-rays as it is more likely to be absorbed by soft plant tissues.

## Results

### Diameter around the scanned portion of the plant stem

**Figure 1.**
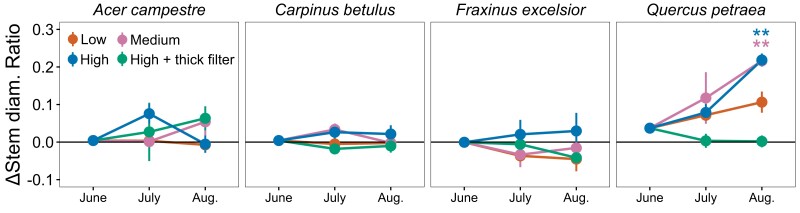
Differences to the control of the diameter ratio (Δstem diam. Ratio; mean ± Se, *n* = 4), i.e. diameter above over the one in the middle of the scanned zone. Stars denote significant differences from the control for a given campaign (*<0.05, **<0.01, and ***<0.001).

For *sessile oak*, we observed significantly higher values of stem diameter ratio (i.e. the ratio between above and the middle of the scanned zone) for the medium and high-energy treatments compared to the control (*P* < 0.001; Fig. 1 and Supplementary Table S1) in August. For this species, whereas the stem diameter ratio of the control trees was decreasing with time (Supplementary Fig. S1), as expected for a tree with stem diameter becoming thinner with height during the growth process, the ratio for trees exposed to medium- and high-energy levels progressively increased, suggesting a disrupted radial growth around the scanned area, i.e. stem deformation. In contrast, the stem diameter ratio was close to 1 for *hedge maple*, *European hornbeam*, and *ash*, suggesting no stem deformation resulting from the X-ray treatments (Supplementary Fig. S1 and Table S1).

### Stem morphology at the scanned location

**Figure 2.**
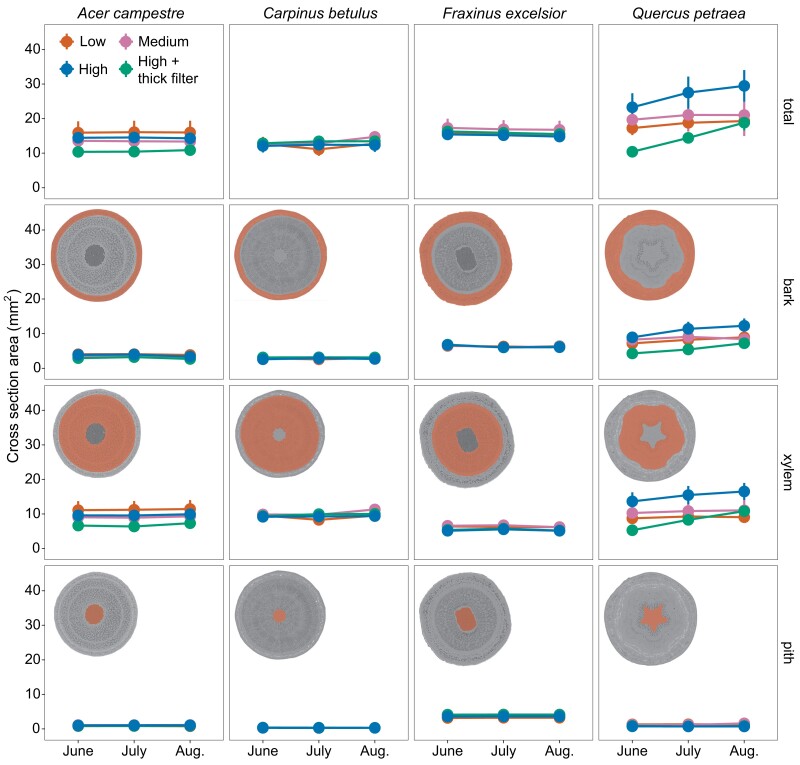
Stem morphology from µCT images: cross section area (mean ± Se, *n* = 4) for different stem tissues (total stem cross-sectional area, bark, xylem, pith). No significant differences were found between the treatments for each date. The embedded pictures show the different tissues area delimitations for one given stem cross section per species.

Across all species, no significant differences between treatments were found in the different tissue areas on the stem cross sections of X-rayed trees (Fig. 2; *P* > 0.05). Pith area was near constant during the 3 mo for all species, as well as the other tissues except for *sessile oak* whose xylem and bark area show a slight upward trend over these 3 mo (statistically similar for all treatments).

### Photosynthetic assimilation and Chl content

**Figure 3.**
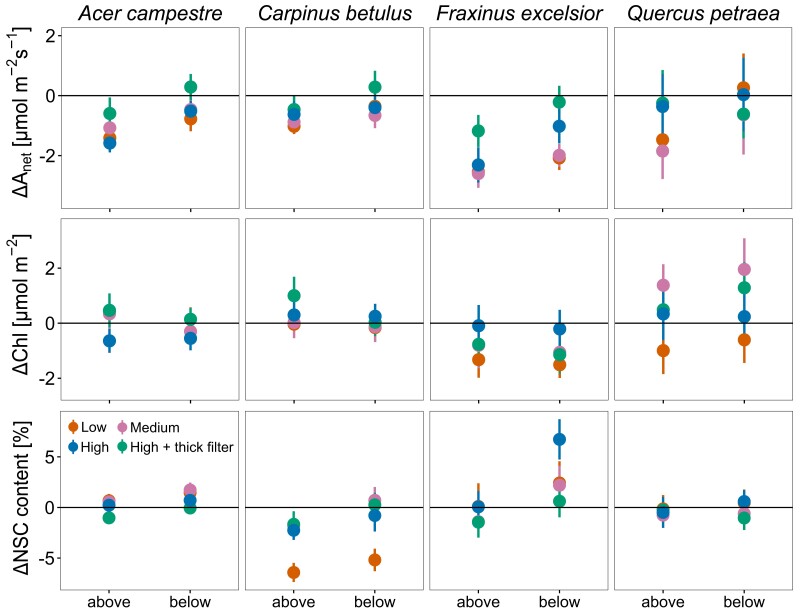
Differences (Δ) to the control of the net assimilation rates *A*_net_, Chl content, and NSC content in the leaves below and above the scanned zone for all four species. No significant differences were found between the control and the treatments. Points indicate means ± Se (*n* = 4 trees).

Overall, the seasonal variations of *A*_net_ and Chl were similar for all treatments (*P* < 0.001 for all species; Supplementary Fig. S2). Results are, therefore, presented by combining data from all 3 campaigns (Fig. 3). No differences in *A*_net_ and Chl between X-rayed trees and the control were found (Fig. 3 and Supplementary Table S2), indicating that the X-ray treatments had no impact for all species (*P* > 0.05; Fig. 3 and Supplementary Table S2). The leaf position did not impact Chl, but *A*_net_ was significantly higher below than above the scanned zone for all species, even for control trees (from +0.53 to +0.83 on average; *P* = 0.004, 0.017, 0.026, and 0.002 for *European hornbeam*, *hedge maple*, *sessile oak*, and *ash*, respectively; Supplementary Table S2 and Fig. S2). A significant treatment × campaign interaction was found for *ash* Chl but did not result in any significant differences from the control (Fig. 3 and Supplementary Fig. S2 and Table S2).

### NSC content

Across all species, no significant differences were found in the leaf NSC content of X-rayed trees compared to control ones (Fig. 3 and Supplementary Table S2; *P* > 0.05) except for *hedge maple* that showed higher NSC in the medium- and low-energy treatments compared to the control during the first campaign, i.e. before any X-ray irradiation. A significant treatment × campaign interaction was also found for *ash* (*P* < 0.001) but did not result in significant differences from the control. The leaf location effect was significant for *European hornbeam*, *hedge maple*, and *ash* (Supplementary Table S2 and Fig. S2) with higher NSC below than above the scanned area, but it was independent of X-ray treatments (Supplementary Fig. S2). Throughout the season, NSC concentrations were progressively reduced in all species apart from *sessile oak* that showed the highest values in July (Supplementary Fig. S2).

### ^13^CO_2_ pulse labeling

**Figure 4.**
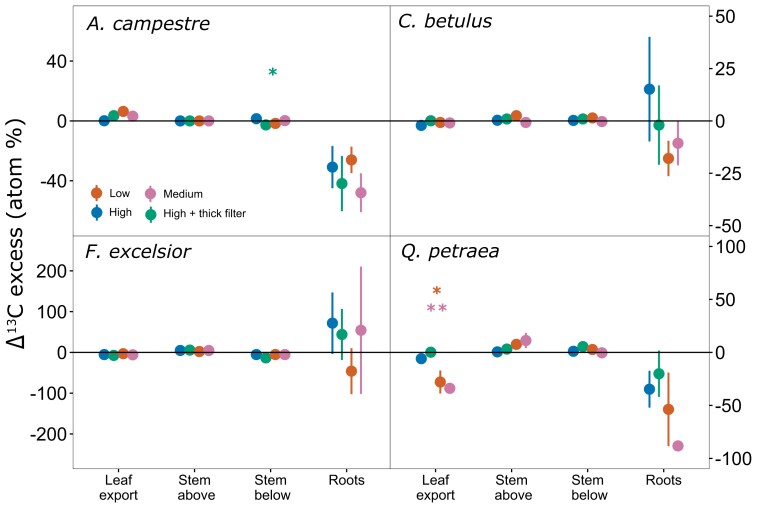
Carbon isotopic (^13^C) excess (mean ± Se, *n* = 4) of X-ray-treated trees compared to the control. Leaf export is the depletion in ^13^C excess in the leaf from Day 1 to Day 6 after labeling (in %), whereas the other tissues were measured 6 d after labeling. Stem and root values were corrected with a factor taking into account the difference in assimilation rate between X-rayed and control trees during the labeling. Stars denote significant differences from the control for a given sample (*<0.05, **<0.01, and ***<0.001). Note the different scales for the different species.

The ^13^C-tracer was found in the roots of all species for all treatments (Supplementary Fig. S3), suggesting functional phloem tissues allowing C transport. Differences in ^13^C excess between X-rayed trees and the control were not significantly different from 0, indicating that the X-ray treatments did not modify the C transport (Fig. 4 and Supplementary Table S3). The amount of assimilated ^13^C that was exported from the leaves to the other parts of the plants was the same for X-rayed trees as for control ones for *hedge maple*, *European hornbeam*, and *ash* (values close to 0). For *sessile oak*, the exported leaf ^13^C was proportionally lower for the low- and medium-energy treatments. Nevertheless, all trees had similar ^13^C excess in the roots compared to the control. For all species, the stem ^13^C excess was not significantly different from the control trees, except for *hedge maple*, which showed significantly lower ^13^C excess in the stem below the X-rayed portion for the high-energy + thick filter treatment. No significant treatment effects were found for *European hornbeam* and *ash* (Fig. 4 and Supplementary Table S3).

## Discussion

Microcomputer tomography is a fairly recent nondestructive imaging method allowing rapid development in our understanding of plant embolism formation. Yet, the current state of research does not allow us to know whether X-rays alter fundamental physiological functions in plants. Overall, no impacts were found on plant physiology and growth except for 1 species, suggesting we cannot completely exclude adverse impacts over the long term but should not expect rapid consequences for plant functions. Indeed, for all studied tree species (*ash*, *European hornbeam*, *sessile oak*, and *hedge maple*) and independently of leaf location, X-ray exposure at low-, medium-, and high-energy levels had no noticeable impact on *A*_net_, NSC, and Chl (Fig. 3). Only the temporal factor (campaign) exerted a significant influence on *A*_net_, Chl, and NSC contents, reflecting the inherent physiological response to the seasonal climatic variations also observed in the control trees (Supplementary Fig. S2). X-rays further had no impacts on the stem diameter growth for *hedge maple*, *ash*, and *European hornbeam* (Fig. 1), and coherently, we observed no impacts on the ability to transport C from the canopy to the roots (Fig. 4). In contrast, *sessile oak*, which showed significant stem deformations around the scanned zone when exposed to medium- and high-energy levels, had similar ^13^C in the roots as the control, suggesting that the plants were still able to transport C (Fig. 4). Moreover, although not significant, we observed slightly less C export from leaf, lower ^13^C excess in the roots, and marginally elevated ^13^C excess in the stem above the scanned zone for the low- and medium-energy treatments in *sessile oak* (Fig. 4). Dry biomass at the end of the experiment (leaves, twigs, and roots) was not significantly affected by the treatments (Supplementary Fig. S4) and do not appear to have influenced the ^13^C excess results.

**Figure 5.**
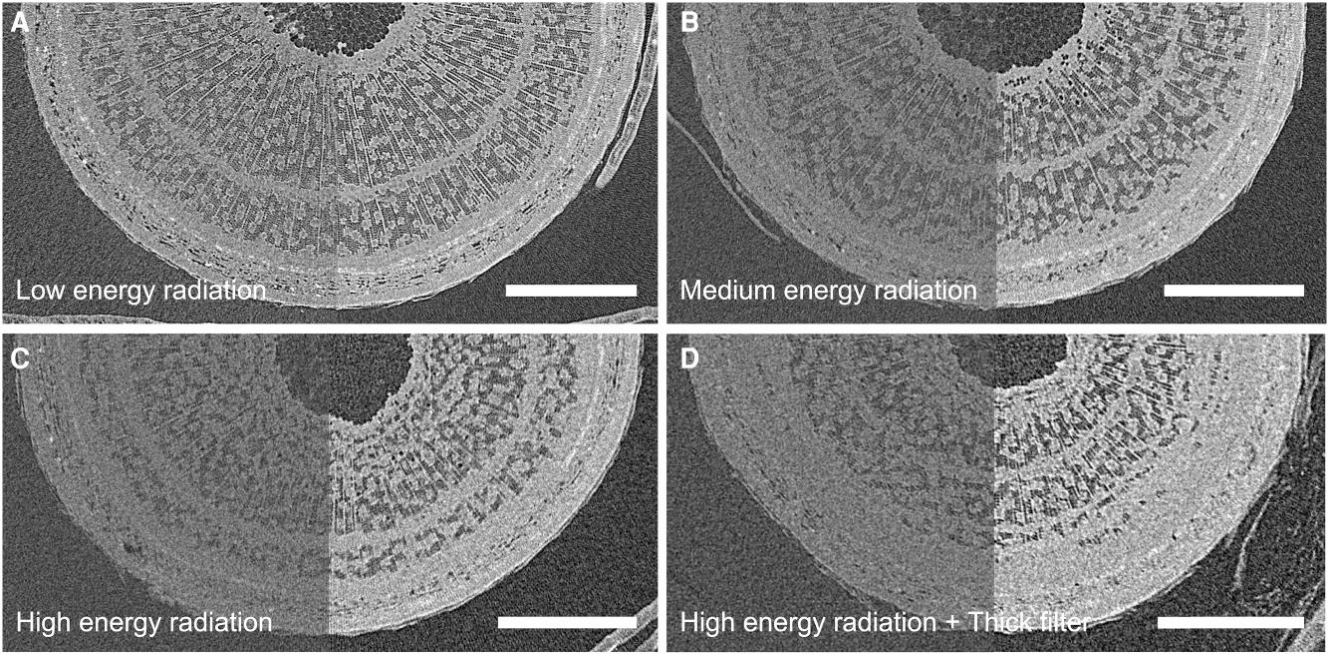
Resulting image quality for each of the four X-ray treatments using μCT scanner. The figure shows examples of images resulting from the X-ray scans. Each stem belongs to a different hedge maple tree. For each image, the left part shows the raw image and the right part is the resulting image after computing the median image over 11 stack images with enhanced contrast. The best contrast and image sharpness can be observed for low-energy radiation (image **A**), with a slight progressive degradation as the energy level and filter thickness increase (see image **B** for medium energy radiation, image **C** for high-energy radiation, and image **D** for high-energy radiation with thick filter). These differences are also visible in the computed median images (which are supposed to reduce noise). White bars represent 1-mm scales.

The high stem diameter ratios found for the medium- and high-energy radiation treatments for *sessile oak* (Fig. 1) could be caused by damaged DNA stimulating cell reproduction above the scanned zone (Petruzzellis et al. 2018), thereby contributing to enhanced growth at this location. The μCT images resulting from the scans could not give information about it, as the singular growth is assumed to happen above the scanned zone—outside the field of μCT images—but they were used to monitor total stem cross section, bark, xylem, and pith areas at the scan location and showed no differences between the scanned trees (Fig. 5). Moreover, *sessile oak* was the only species exhibiting a slight increase in xylem and bark areas over the 3 mo, suggesting that radial growth may be reduced rather than completely stopped at the scanned location. It remains to be understood whether the extra-tissues above include xylem and whether they participate in water transport. Indeed, the 3D reconstruction of a deformed tree that had been scanned at the μCT platform before the current study shows that some of the outgrowth on the scanned area and below had a reduced xylem vessel density compared to the trunk that developed 1 yr after the irradiation (Supplementary Fig. S5). This 3D reconstruction also clearly illustrates the near cessation of growth at the exact location of the scan; i.e. 1 growth ring is missing for each year passed since the irradiation. The radial growth cessation suggests the cambium cells are likely to be damaged locally. These observed anatomical alterations offer valuable considerations regarding potential long-term impacts uncovered in this study's results. The locally reduced stem diameter could act as a bottleneck for the tree by limiting the conductive area of the xylem, and eventually distort experiments aimed at studying the resistance of trees to drought. Indeed, the ability of trees to survive drought is linked to the survival of meristematic cells, both primary and secondary, such as cambium cells, involved in the development of vascular tissues (Mantova et al. 2022). Nevertheless, the phloem tissues that probably received equal or higher doses of X-ray seemed to maintain their ability to transport C. Two main mechanisms can explain the negative impacts of X-ray: direct damage to specific targets within the cell and indirect damage to the cell as a result of ionization of water or other molecules in the cell (Whaites and Drage 2002). Nevertheless, more work would be needed to fully understand the drivers of growth cessation in *sessile oak*. The density of the wood of each species could explain some species-specific reactions of trees to X-rays. However, according to the Tree functional attributes and Ecological database (https://apps.worldagroforestry.org/treedb/), the species show very low differences in their wood density (*ash*: 0.6080 g/cm^2^; *sessile oak*: 0.6332 g/cm^2^; *European hornbeam*: 0.6653 g/cm^2^; *hedge maple*: 0.6150 g/cm^2^). Hence, any distinctions are more likely connected to other parameters.

Our design included 3 different doses, going from 21 Gy (low), 42 Gy (medium and high with thick filter), to 66 Gy (high) after 3 scans. While the impacts on radial growth in *sessile oak* were strongest as the X-ray doses increased (Fig. 4), the other species showed no response and physiology was not impacted in the 4 species. Interestingly, impaired radial growth was not observed for the high-energy + thick filter treatment, although it represents the same applied dose as the medium-energy treatment. This finding supports the hypothesis that, for the same dose, higher-energy radiations do not have a greater impact on tree anatomy, when low-energy radiations are filtered out. However, we still lack consistency in the results to determine whether energy levels may be more important than the applied dose in anticipating possible damage to plants during in vivo μCT. Using a 0.5-mm aluminum filter instead of a 0.1-mm filter appears to have potentially prevented impacts on the trunk's anatomy. However, Stupian (2007) had not observed any effect on radiation dose using a 1.6-mm aluminum filter. In our case, the filter slightly decreased both the applied dose and the resulting image quality (Fig. 5), which confirms the importance of deliberating on the choice of filter, striking a balance between preventing impacts, and maintaining image quality.

Our findings confirmed the noninvasive nature of in vivo μCT scans on plant physiology. Yet, they also indicate potential adverse impacts on the anatomy of the scanned trunks, regardless of the energy levels and the doses administered by the UltraTom by RX-Solutions. Previously published data had shown that multiple μCT scans could disrupt fundamental cellular functions and processes (Petruzzellis et al. 2018). Their experiments were performed at the SYRMEP beamline (Elettra Sincrotrone Trieste, www.elettra.trieste.it) and included 2 silicon filters (0.5 mm each), leading to an average X-ray source energy of 25 keV, resulting in an entrance dose of 47 mGy s^−1^. In comparison, our treatments ranged from 5 to 34 mGy s^−1^ (Table 1). In their study, they also used synchrotron-based μCT, whereas we used a laboratory-based μCT system, which may result in different X-ray impacts. As the technology used to produce X-rays differs, this often results in higher radiation intensities and shorter scan times for synchrotrons. Hence, the results of this study may not be interpolable to synchrotron experiments, as the ranges tested for the various parameters may not correspond. The relevance of any range of energy levels and doses is worth examining in future studies for different machines allowing in vivo μCT.

**Table 1.** The 4 energy levels X-ray treatments that have been applied to the trees by μCT scanning them monthly for a period of 3 mo

Treatment (energy levels)	Low	Medium	High	High + thick filter
Tube voltage (kV)	40	60	80	80
Current (μA)	108	115	88	88
Frame rate (FPS)	0.7	2.1	2.85	2.85
Average img	1	2	2	2
Time (min)	24	16	11	11
White value	13,500	13,800	13,800	13,800
Aluminum filter (mm)	0.1	0.1	0.1	0.5
Focus tube type	Nano	Micro	Micro	Micro
Absorbed dose to water (Gy) Co-60 equivalent (per scan)	10	19	30	19
Absorbed dose to water for X-ray (Gy) (per scan)	8	14	22	14
Norm. absorbed dose to water for X-ray (mGy s^−1^)	5	14	34	22

Control trees experiencing no X-ray treatment functioned as a fifth treatment.

## Conclusion

In this study, we showed that tree physiology is not responding to the energy levels and doses of X-ray after 3 monthly µCT scans. However, X-rays led to the cessation of stem radial growth and stem deformations for *sessile oak*, although a ^13^C labeling confirmed that the phloem was still operational at the end of the experiment. These results suggest that it is unnecessary to adjust scan parameters on laboratory-based µCT similar to the one used in the study to preserve trees from X-rays. Consequently, spectrum qualities can simply be selected to maximize image quality within the range of our tested parameters. We can also reasonably confirm the reliability of all studies using µCT on living trees when carried out within a fairly short term, including different tree species and repeated physiological measurements. Hence, the noninvasive nature of μCT makes it particularly interesting to monitor plants by scanning them several times, to analyze drought and recovery cycles (Choat et al. 2015).

So far, the majority of published work does not extend the experiments involving μCT scans to more than a few weeks (Choat et al. 2015; Knipfer et al. 2015; Rehschuh et al. 2020) even when repeating the scan up to 8 to 10 times (Brodersen et al. 2013; Choat et al. 2015; Charrier et al. 2016; Knipfer et al. 2017, 2019; Peters et al. 2020). However, it is essential to keep things in perspective as the induced damage to plants may depend on the time between exposition (Whaites and Drage 2002). Some studies extended the observations of scanned trees up to 6 mo or even 1 yr (Knipfer et al. 2019; Li et al. 2020), which goes beyond our 3-mo study. In these cases, one should consider that there is a potential lag time between the X-ray treatment and anatomical and plant physiological effects, which our study may have not observed. Caution should also be taken for any µCT setup that would differ from the one used in this experiment. Important parameters to consider are as follows: the time between each scan, source-to-sample distance (Zappala et al. 2013), total X-ray doses, and duration of the physiological measurement periods—specifically if related to stem hydraulics. In this way, we emphasize the importance of providing precise information on the conditions of use of µCT techniques when used with living trees. In conclusion, our data suggest that monthly laboratory-based µCT scans do not impact the plant physiology and the C transport over 3 mo, but it may be invasive for longer exposure times during drought experiments.

## Materials and methods

### Experimental design

The experiment was carried out between June and September 2021 and consisted of repeatedly applying four X-ray treatments (as detailed below) and monitoring physiological traits before each scan. Four tree species were selected on the drought tolerance spectrum (based on their respective *P*_50_ values, i.e. as often in drought experiments using µCT): ash (*P*_50_ = −2.8), sessile oak (*P*_50_ = −3.5 MPa), European hornbeam (*P*_50_ = −3.8), and hedge maple (*P*_50_ = −5.7) (Choat et al. 2012). While we did not expect plants’ *P*_50_ to directly impact their response to X-rays, we wanted to assess if trees with contrasting drought tolerance responded consistently so that in vivo µCT can be used reliably in manipulative drought experiments. The seedlings of 2-yr-old trees were potted in June 2021 and remained outdoors on the EPFL campus (Lausanne, Switzerland, yearly precipitation sum of 1,000 mm and mean air temperature of 11 °C in 2021, MeteoSwiss) in an uncovered area until the end of the experiment. Throughout the experiment, the plants were continuously irrigated using automatic drip irrigation.

At the start of the experiment, the mean tree heights were 65 ± 7, 67 ± 6, 50 ± 13, and 58 ± 8 cm for ash, European hornbeam, sessile oak, and hedge maple, respectively. As the height of the trees was limited by the space available in the µCT scanner, it was important that the trees were not too tall at the time of the measurements. The experimental design included 4 repetitions per treatment and species plus control trees, leading to 60 individual trees receiving different X-ray treatments and 20 control trees (receiving no X-ray during the experiment). In total, 80 trees were used.

**Figure 6.**
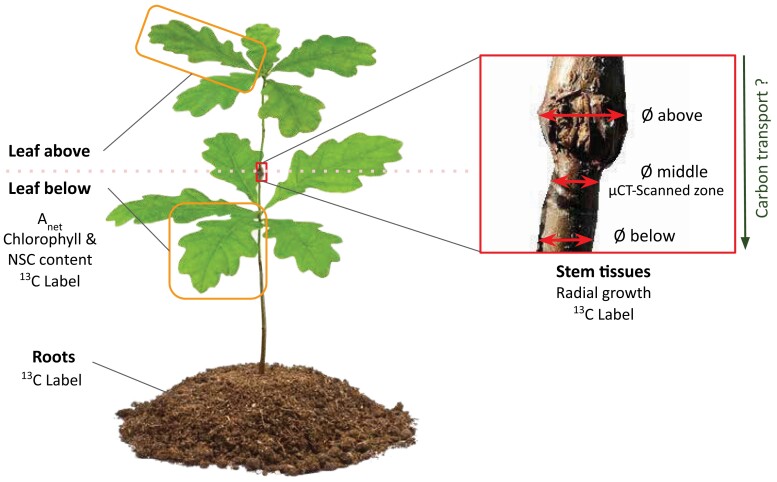
Schematic representation of the measurement design. The inset is the µCT-scanned portion of the stem (about 1-cm-long portion) where stem diameter (Ø) was tracked above, below, and at the scanned location. Leaves above and below the scanned zone were monitored during the whole experiment for assimilation rates (*A*_net_), Chl, and NSC contents. At the end of the experiment, a ^13^C label was applied to tree canopies to determine carbon transport, and ^13^C was measured in the leaves, stem, and root tissues.

At the beginning of the experiment, a 1-cm-long portion of stem was selected and marked with adhesive tape to identify the portion of the stem that was going to receive the monthly X-ray radiations (Fig. 6). This area was chosen such that there was at least 1 branch below for the physiological measurements (i.e. made on leaves on either side of the scanned area). We selected and marked 1 mature leaf above and below the scanned zone for repeated gas exchange and Chl content measurements (see below).

### Stem radial growth around the scanned location

Prior to each scan, a digital caliper was used to monitor diameter changes below, in the middle of the scanned zone, and above (Fig. 6). The stem diameter ratio was calculated as the measured stem diameter above the scanned zone divided by the one in the middle.

### Stem morphology at the scanned location

The cross-sectional area of different stem tissues was measured on the images resulting from μCT scans: bark, xylem, pith, and total stem cross section being the sum of the latest. Data were collected 3 times (at each scan) but are not available for control trees as they were never μCT scanned. First, the median image of 10 cross-sectional images was computed to reduce noise and enhance contrast and edge clarity. The areas were then delimited manually and measured using ImageJ 1.54f. The pith was easily defined as the area bounded by the inner xylem (small nonconductive vessels close to the pith, clearly visible on the μCT images as vessels filled with air stand out in black with strong contrast) and/or evident change in tissue structure. The boundary of the xylem area was drawn where the slice displayed a change in structure and/or white contrasting elements (corresponding to the phloem having a greater density than the water or surrounding tissues). The bark area was defined as the area remaining once the pith and xylem area had been traced.

### MicroCT scans

Four X-ray μCT treatments were applied monthly in July, August, and September on each plant (Table 1) using a μCT scanner (UltraTom, RX Solutions, Chavanod, France). The highest energy of X-ray photons generated in a tube was determined by the product of the tube voltage; 3 levels of voltage were chosen (low, 40 kV; medium, 60 kV; and high, 80 kV) consequently modifying not only the maximum energy level reached by the photons but also the whole spectrum. A fourth treatment of high voltage with a thicker filter (in aluminum of 0.5 mm vs. 0.1 mm in the classic settings) was added to minimize the impacts from low-energy X-rays. Indeed, filters produce a narrower-spectrum X-ray beam by attenuating the lowest X-rays (Stupian 2007). We first identified the resolution required to obtain sharp images (4 to 6 *μ*m based on previous scans performed on the same platform), which lead us to choose the microfocus tube of the UltraTom μCT system (that offers the adequate range of voltages). The nanofocus tube was used only for the low-energy radiation treatment, as it offers more stable conditions than the microfocus tube with this voltage.

Given these different energy levels, the rest of the scan parameters, including the current, scan duration, etc., were adapted to always generate sharp and usable images (by keeping a similar white value on the screen). The goal was to compare scan parameters that are usable in practice to assess xylem embolism to optimize the parameters used without compromising image quality. However, even while adjusting the parameters to have similar white values for each treatment, we observed that low-energy radiation scans gave sharper images than higher-energy ones (Fig. 5), most probably for X-ray physics reasons. Indeed, differences in image quality can be attributed to the absorption characteristics of soft tissues, such as the stems of young trees, which theoretically absorb low-energy radiations more effectively.

The corresponding doses (in Gy) were measured using thermoluminescent dosimeters (TLDs) in collaboration with the CHUV Institute of Radiophysics (Lausanne, Switzerland). The TLDs were scanned under the same conditions as the experimental trees (Supplementary Fig. S6). We used round TLD-100 LiF:Mg, Ti chips (Thermo Fisher, USA) with a diameter of 4.5 mm and a thickness of 0.9 mm. They were individually calibrated in terms of absorbed dose to water and traceable to the primary standard of METAS (Stucki Muench and Quintel 2002). The standard reading procedure is described in Jaccard et al. (2017). The TLDs were calibrated for Cobalt 60 (Co-60)—one of the most used sources of radiation—such that an energy correction factor needs to be computed to take into account the differences in energy between Co-60 and the X-ray beams from our study. The energy correction factors were computed using calculated half-value layer and mean X-ray energy and were found to be between 0.71 and 0.78. An average correction factor of 0.75 was used, and regarding the slight relative bias induced by the range of beam parameters, we decided to integrate this effect within the uncertainty evaluation. The absorbed dose-to-water values are, therefore, associated with a standard uncertainty of 11%.

### Photosynthetic assimilation and Chl content

Prior to each scan, measurements of the net light-saturated photosynthesis (*A*_net_, mmol m^−2^ s^−1^) were conducted on one leaf above and below the scanned zone (Fig. 6) with 2 LiCor LI-6800 (LiCor Inc., Lincoln, USA) equipped with a 2-cm^2^ fluorescence leaf chamber. Each leaf was clipped in the cuvette, set to ambient air temperature and relative humidity. The measurements were done at saturated light intensity (1,500 *μ*mol m^−2^ s^−1^ PAR), CO_2_ concentration of 400 ppm, and flow at 500 *μ*mol s^−1^. While 1,500 *μ*mol m^−2^ s^−1^ is generally above the ambient light conditions, using this standard light value during gas exchange measurements ensures cross-comparison with other studies and light saturation of the trees. The measurements for each campaign were conducted during 2 consecutive days with similar climatic conditions. Chl concentration (µmol m^−2^) was measured on the 2 same leaves during the whole experiment (the same leaves as those used for *A*_net_ measurements) with a Chl concentration meter (MC100; Apogee Instruments Inc., Logan, UT, USA).

### NSC content

NSCs are defined here as low-molecular-weight sugars (glucose, fructose, and sucrose) and starch. Before each scan, we sampled 1 leaf above and below the scanned area, which was dried at 60 °C until a stable weight was achieved and then ground to a fine powder. NSCs were analyzed following the protocol described by Wong (1990), adapted according to Hoch et al. (2002). Briefly, 10 to 12 mg of ground material was boiled in 2 ml of distilled water for 30 min. The total amount of NSC was measured by incubating 500 *μ*l of the extract (“Total NSC,” including sugars and starch) with a fungal amyloglucosidase from *Aspergillus niger* (Sigma-Aldrich) for 15 h at 49 °C to digest starch into glucose. After centrifugation, an aliquot of 200 *μ*l was treated with Invertase and Isomerase from baker's yeast (*Saccharomyces cerevisiae*, Sigma-Aldrich, St Louis, MO, USA) to degrade sucrose and convert fructose into glucose (“Sugars”). Sugars and total NSC were determined at 340 nm in a 96-well microplate photometer (HR 7000; Hamilton, Reno, NE, USA) after enzymatic conversion of glucose to gluconate-6-phosphate (hexokinase reaction, hexokinase from Sigma Diagnostics, St Louis, MO, USA). Pure starch and glucose, fructose, and sucrose solutions were used as standards, and standard plant powder (Orchard leaves; Leco, St Joseph, MI, USA) was included to control the reproducibility of the extraction. NSC concentrations are expressed on a percentage dry matter basis. Because all samples were run in a single laboratory with no change in protocol during the laboratory processing of samples, issues with comparing results across methods or laboratories were obviated (Quentin et al. 2015).

### ^13^CO_2_ pulse labeling

^13^CO_2_ labeling was conducted on September 15, 2021 (after the last μCT scans) on all trees to track C transport from the canopy to the roots. All trees were labeled, but the ones that had been scanned were stripped of all leaves below the scanned area to restrict C uptake to the portion above the scanned stem location. The trees were subjected to a controlled incubation (Supplementary Fig. S7) in a sealed greenhouse with ^13^CO_2_ injection from 12:00 to 18:00 CEST. The CO_2_ concentration in the greenhouse was tracked during the labeling using an open-path CO_2_ analyzer (GMP343, Vaisala, Finland). The gas-tight low-density polyethylene labeling greenhouse permitted about 85% transmittance of photosynthetically active radiation. ^13^C was applied as ^13^CO_2_ to the aboveground parts of the plants by pulse labeling. One fan was installed in the center of the chamber to ensure the even distribution of ^13^CO_2_. The ^13^CO_2_ was released by mixing it with H_2_SO_4_. The glass bottle (containing 10 g of Na_2_^13^CO_3_ and 10 g of nonisotopically enriched analog Na_2_CO_3_) was connected to the inside of the sealed chamber through plastic tubes. We introduced 5 to 15 mL of 1 m H_2_SO_4_ to the glass bottle using a syringe at 5-min intervals over a period of 6 h. In total, 290 mL of 1 m H_2_SO_4_ were added to 20 g of Na_2_^13^CO_3_ and 20 g of Na_2_^12^CO_3_ (we added an additional shot of 10 g of ^13^C and 10 g of ^12^C as the Na_2_^13^CO_3_ and H_2_SO_4_ had reacted completely).

At the end of the day, 1 to 2 leaves were collected from each tree to evaluate the success of the ^13^CO_2_ assimilation by the plants. Six days later, the trees were excavated, and new samples were collected: one leaf, one portion of the stem above and one below the scanned area, and fine roots. All leaf, stem, and root materials were dried at 60 °C until stable weight was achieved and then ground into a fine powder. The remaining biomass was sorted (leaves, wood, and roots of each tree) and dried before being weighed to further calculate excess ^13^C. The excess ^13^C values in plant compartments were computed following Ruehr et al. (2009). The isotopic ^13^C/^12^C ratio in all samples was expressed in delta notation (‰) relative to the international Vienna Pee Dee Belemnite standard. Thereafter, ^13^C in bulk plant material was converted to atom% as follows:


(1) $$\mathrm{atom}\text{\%}=\frac{100\cdot 0.0111802\cdot \left(\frac{\delta }{1,000}+1\right)}{1+0.0111802\cdot \left(\frac{\delta }{1,000}+1\right)},$$

where 0.0111802 is the standard value for the isotopic ^13^C/^12^C ratio of Vienna Pee Dee Belemnite standard. To calculate excess ^13^C (mg m^−2^) for each organ (i.e. the amount of ^13^C added to the respective organ due to ^13^C labeling), atom% was normalized per organ dry weight (DW) and its natural isotope baseline prior labeling (Ruehr et al. 2009):


(2) excessC13=atom%s−atom%b100⋅B⋅C%100,

where atom%*_s_* is the atom% of the organ sampled, atom%*_b_* is the atom% of the natural unlabeled background for each species and organ, *B* (mg m^−2^) is the biomass DW averaged per species, treatment, and organ (*n* = 10) and referred to the pot ground area (0.02 m^2^), and C% is the percentage of C in the sample. Leaf natural abundance was measured for each tree, as leaf samples were taken before the labeling. Natural abundances of stem tissues and roots were measured once for each species and organ, a few weeks before the labeling on trees of the same age and provenance than the treated trees.

For the stem and root measurements, the values were normalized according to the assimilation rate that took place during labeling (Equation 3). In this way, it becomes possible to determine if the tissues exhibit changed ^13^C levels as a result of declined C transport or uptake.


(3) $$\begin{array}{r} \mathrm{Normalized}\;\mathrm{leaf}\;\mathrm{export}=\frac{\mathrm{Lea}f_{\mathrm{Day}1}\;\mathrm{Excess}-\mathrm{Lea}f_{\mathrm{Day}6}\;\mathrm{Excess}}{\mathrm{Lea}f_{\mathrm{Day}1}\;\mathrm{Excess}}\cdot 100. \end{array}$$

Finally, to evaluate whether the C depletion in the leaves was consistent between X-rayed trees and the control regardless of the amount of C assimilated during the labeling, the leaf exported ^13^C excess (from Day 1 to Day 6) was computed according to Equation (4).


(4) Corrected excessC13=ExcessC13 Coeffsp,trtwhere Coeffsp,trt=Lsp,trteafDay1ExcessLsp,ControleafDay1Excess.

### Statistical analyses

To assess the impact of the different X-ray treatments on the measured parameters (*A*_net_, Chl, NSC in leaves, and stem diameters ratio), we used linear mixed-effects models. The X-ray treatment (control, low, medium, high, and high + thick filter), the measurement campaign (campaigns), and the location of the leaf (above vs. below the scanned zone) were included as fixed effects, as well as all their interactions, and the tree (repetition) was considered as a random effect. The location was also included as a fixed effect for stem diameter measurements around the scanned area (below, middle, and above). Regarding ^13^C excess in the leaves, stem, and roots, we included in the linear mixed model the treatment, species, and their interaction as fixed effects, and tree was the random effect. Post hoc analysis was performed with Tukey's honestly significant difference (HSD) test, with false discovery rate (FDR) correction for multiple testing. For the stem morphology measurements resulting from μCT images, the linear mixed-effects model included treatment as a fixed effect (low, medium, high, and high + thick filter—control was not part of these measurements as control trees were never μCT scanned) and tree (repetition) was considered as a random effect. Treatment effects were then tested for every species, scan (June, July, and August), and tissue area (total stem cross section, bark, xylem, and pith). All statistical analyses were performed with R v.4.0.4 (RCore Team, 2021).

## Supplementary Material

kiae285_Supplementary_Data

## Data Availability

Data used in this manuscript will be available from the Dryad Digital Repository after acceptance. Data supporting the findings of this study are also available from the corresponding author, L.M.

## Supplementary data

The following materials are available in the online version of this article.

**Supplementary Figure S1.** Diameter ratio above the scanned zone over the one below (mean ± Se, *n* = 4).

**Supplementary Figure S2.** Mean (±Se, *n* = 4) net light-saturated assimilation (*A*_net_), Chl content, and NSC of leaves.

**Supplementary Figure S3.** Mean (±Se, *n* = 4) carbon isotopic (^13^C) excess in the different tissues (roots, leaf, and stem above and below the scanned zone) measured 6 d after the labeling.

**Supplementary Figure S4.** Dry biomass (mean ± Se, *n* = 4) of different tissues (leaves, roots, and twigs including stems) sampled 6 d after the labeling.

**Supplementary Figure S5.** Images from a 3D reconstruction (using Avizo 2021 software) of a 5-yr-old European hornbeam tree stem deformed by X-ray radiation (resulting from μCT scan).

**Supplementary Figure S6.** Setup at the PIXE µCT platform (EPFL, Lausanne, Switzerland).

**Supplementary Figure S7.** Experimental setup used for ^13^CO_2_ labeling.

**Supplementary Table S1.** Statistical analyses of the stem diameter ratio.

**Supplementary Table S2.** Statistical analyses of physiological measurements (*A*_net_, Chl, and leaf NSC content).

**Supplementary Table S3.** Statistical analyses of the ^13^CO_2_ pulse labeling results.

## Dive Curated Terms

The following phenotypic, genotypic, and functional terms are of significance to the work described in this paper:

stem AmiGo: PO:0009047

leaf AmiGo: PO:0025034
