# Supplementary material for: In vivo X-ray microtomography locally affects stem radial growth with no immediate physiological impact
Source: Plant Physiol. 2024 May 17;196(1):153–63. doi: 10.1093/plphys/kiae285 (PMC11491841; doi:10.1093/plphys/kiae285)
Supplement: kiae285_Supplementary_Data [file kiae285_supplementary_data.pdf]

Supplementary Data

Supplementary Table S1: Statistical analyses of the stem diameter ratio. Summary of the ANOVA test of the linear mixed-effects model (F- and p-values) testing the effects of treatment, campaign, species (CB: *Carpinus betulus*, AC: *Acer campestre*, QP: *Quercus petraea*, and FE: *Fraxinus excelsior*), and their interactions on the diameter ratio, i.e. the diameter above the scanned zone over the one in the middle of scanned zone). Significant effects (p ≤ 0.05) are highlighted in bold.

Factor	Species	F-value (p-value)
	CB	
Treatment		1.86 (0.1346)
Campaign		1.19 (0.3138)
Treatment:Campaign		1.08 (0.3954)
	AC	
Treatment		0.79 (0.5391)
Campaign		0.59 (0.5611)
Treatment:Campaign		1.05 (0.4150)
	QP	
**Treatment**		**8.25 (4.703 e-05)**
**Campaign**		**3.52 (0.0382)**
**Treatment:Campaign**		**3.57 (0.0029)**
	FE	
Treatment		1.81 (0.1435)
Campaign		0.88 (0.4230)
Treatment:Campaign		0.81 (0.5958)

Model: lme(diam_ratio ~ treatment*campaign, random=~1|tree, na.action=na.omit)

Supplementary Table S2: Statistical analyses of physiological measurements. Summary of the linear mixed-effects models (F- and p-values) for each species (CB: *Carpinus betulus*, AC: *Acer campestre*, QP: *Quercus petraea*, and FE: *Fraxinus excelsior*) with the interactive effects of treatment (TRT), leaf position (LEAF, i.e., below vs. above irradiated zone), campaign (CAMP). Significant effects (p ≤ 0.05) are highlighted in bold.

Source of variation	Chl	A_{net}	NSC
CB			
TRT	0.26 (0.900)	1.63 (0.217)	2.82 (0.063)
LEAF	2.12 (0.149)	**8.88 (0.004)**	**11.34 (0.0012)**
CAMP	**79.56 (< .0001)**	**52.59 (< .0001)**	**22.92 (< .0001)**
TRT:LEAF	0.88 (0.482)	0.64 (0.638)	0.17 (0.954)
TRT:CAMP	0.63 (0.751)	2.19 (0.037)	1.62 (0.132)
LEAF:CAMP	0.62 (0.541)	0.24 (0.789)	1.27 (0.288)
TRT:LEAF:CAMP	**2.36 (0.025)**	1.69 (0.115)	0.75 (0.651)
AC			
TRT	0.46 (0.766)	1.26 (0.329)	**3.37 (0.037)**
LEAF	**5.44 (0.022)**	**6.02 (0.017)**	**16.16 (0.0001)**
CAMP	**190.24 (< .0001)**	**3.63 (0.031)**	**39.28 (< .0001)**
TRT:LEAF	0.83 (0.509)	1.66 (0.169)	0.32 (0.867)
TRT:CAMP	1.19 (0.314)	1.32 (0.249)	**3.03 (0.005)**
LEAF:CAMP	**15.64 (< .0001)**	0.27 (0.763)	1.21 (0.303)
TRT:LEAF:CAMP	1.19 (0.319)	1.06 (0.399)	0.76 (0.640)
QP			
TRT	1.58 (0.231)	0.24 (0.914)	0.28 (0.891)
LEAF	0.44 (0.511)	**5.23 (0.0250)**	0.33 (0.567)
CAMP	1.93 (0.153)	**76.22 (< .0001)**	**23.22 (< .0001)**
TRT:LEAF	0.08 (0.989)	1.02 (0.402)	0.30 (0.880)
TRT:CAMP	0.38 (0.928)	1.30 (0.255)	1.30 (0.255)
LEAF:CAMP	0.04 (0.965)	1.33 (0.272)	0.36 (0.702)
TRT:LEAF:CAMP	0.63 (0.748)	0.66 (0.726)	0.28 (0.972)
FE			
TRT	0.91 (0.482)	2.35 (0.101)	0.99 (0.442)
LEAF	1.28 (0.262)	**10.45 (0.0018)**	**24.58 (< .0001)**
CAMP	**103.85 (< .0001)**	**17.72 (< .0001)**	**83.00 (< .0001)**
TRT:LEAF	0.04 (0.996)	1.03 (0.398)	1.59 (0.187)
TRT:CAMP	**2.51 (0.018)**	1.94 (0.067)	**3.83 (0.0008)**
LEAF:CAMP	1.17 (0.315)	0.60 (0.553)	0.09 (0.916)
TRT:LEAF:CAMP	0.99 (0.448)	0.26 (0.977)	0.38 (0.931)

*Full model: lme(physiological traits ~ treatment*leaf*campaign, random=~1|tree)*

Supplementary Table S3: Statistical analyses of the $^{13}CO_2$ pulse labeling results. Summary of the linear mixed-effects models (F- and p-values) of treatment (TRT), species (SP) and their interaction on the ^{13}C excess measured in the leaves right after the labelling (LAF) and 6 days later (LAF6), stem tissue above (SABO) and below (SBEL) the µCT scanned zone, and fine roots (FR). All the last were sampled 6 days after labeling. Significant effects ($p \leq 0.05$) are highlighted in bold. The second table highlights the summary for each species (CB: *Carpinus betulus*, AC: *Acer campestre*, QP: *Quercus petraea*, and FE: *Fraxinus excelsior*).

Global model:

Source of variation	LAF	LAF6	SABO	SBEL	FR
TRT	**3.19 (0.044)**	2.61 (0.077)	1.25 (0.331)	1.03 (0.423)	**3.92 (0.023)**
SP	**16.26 (< .0001)**	**17.18 (< .0001)**	**42.62 (< .0001)**	**28.81 (< .0001)**	**3.52 (0.023)**
TRT:SP	**5.87 (< .0001)**	**3.31 (0.002)**	1.95 (0.053)	**3.27 (0.002)**	**3.78 (0.0006)**

*Full model: lme(excess-13C ~ treatment*species, random=~1|tree)*

Treatment effect per species:

Source of variation	LAF	LAF6	SABO	SBEL	FR
CB	**5.59 (0.0058)**	1.76 (0.190)	**3.19 (0.0442)**	2.04 (0.141)	0.15 (0.958)
AC	**4.29 (0.0164)**	**3.32 (0.0389)**	0.34 (0.845)	**10.71 (0.0003)**	1.43 (0.272)
QP	**4.94 (0.0096)**	**3.16 (0.0452)**	2.07 (0.136)	2.56 (0.0813)	**5.37 (0.0069)**
FE	**70.26 (< .0001)**	**5.79 (0.0058)**	0.56 (0.694)	2.21 (0.117)	2.81 (0.0637)

Model: lme(excess-13C ~ treatment, random=~1|tree)

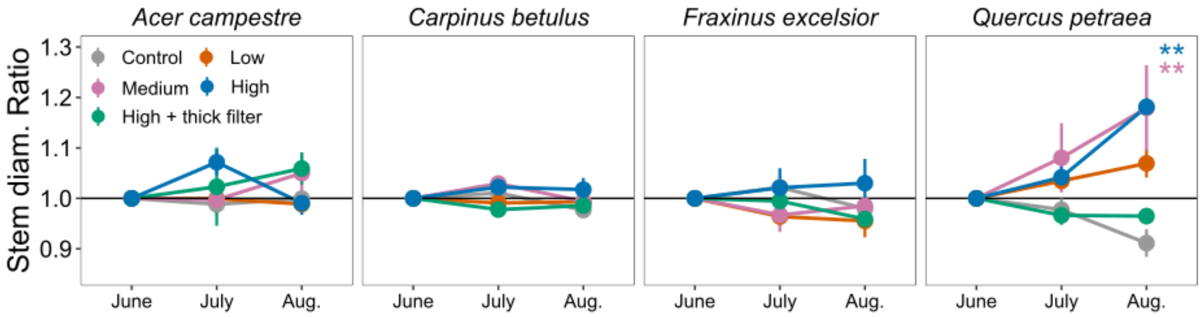

Supplementary Figure S1. Diameter ratio above the scanned zone over the one below (mean ± SE, n=4). Values above 1 indicate larger stems above the scanned zone while negative values indicate a normal situation with a stem diameter decreasing with height for all four species. Stars denote significant differences from the control for a given date (*<0.05, **<0.01, ***<0.001).

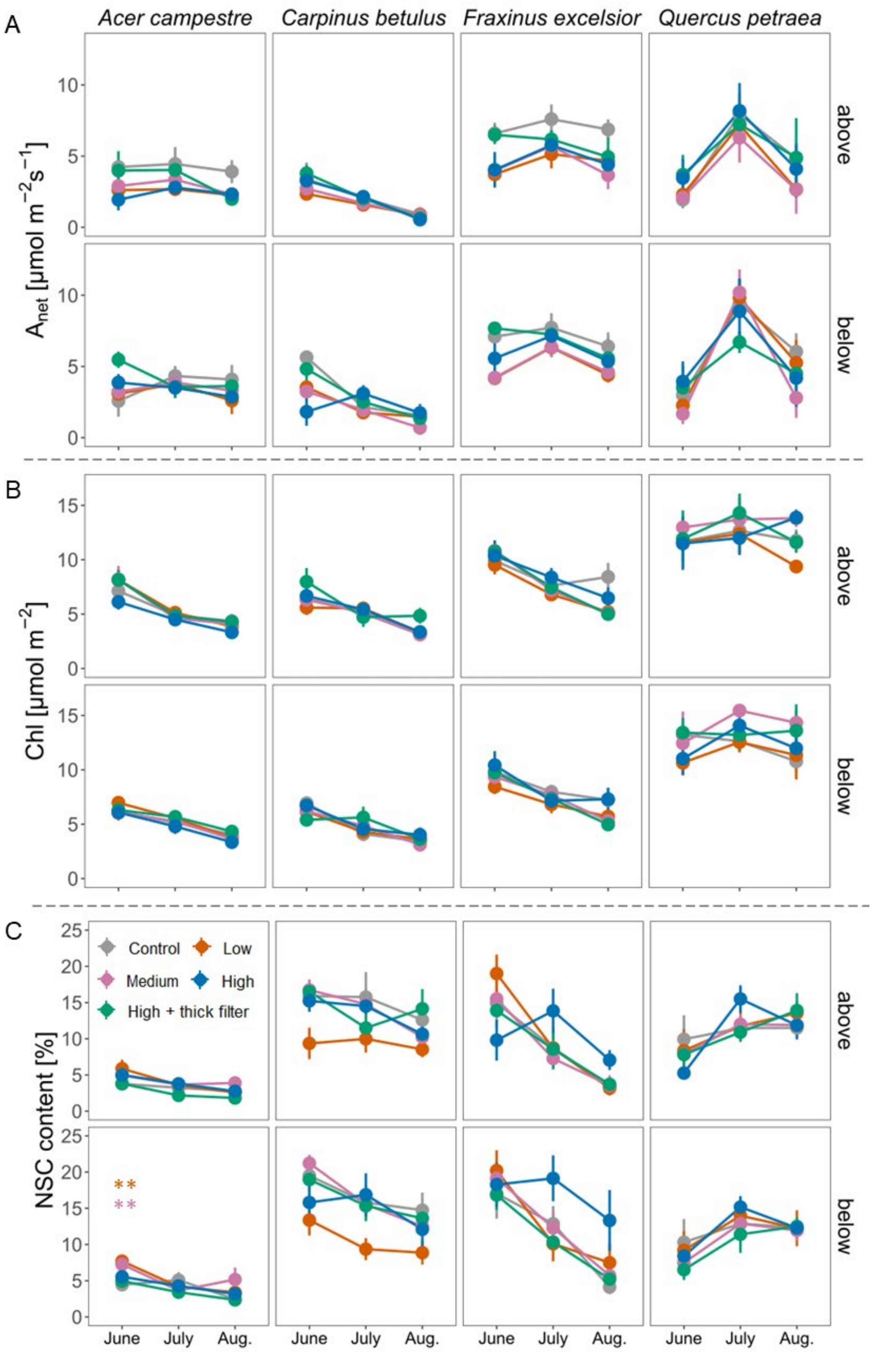

Supplementary Figure S2. Mean (±SE, n=4) net light-saturated assimilation (A_{net}), chlorophyll content (Chl), and non-structural carbon content (NSC) of leaves below and above the scanned zone for all

species and treatments. Stars denote significant differences from the control for a given date (*<0.05, **<0.01, ***<0.001).

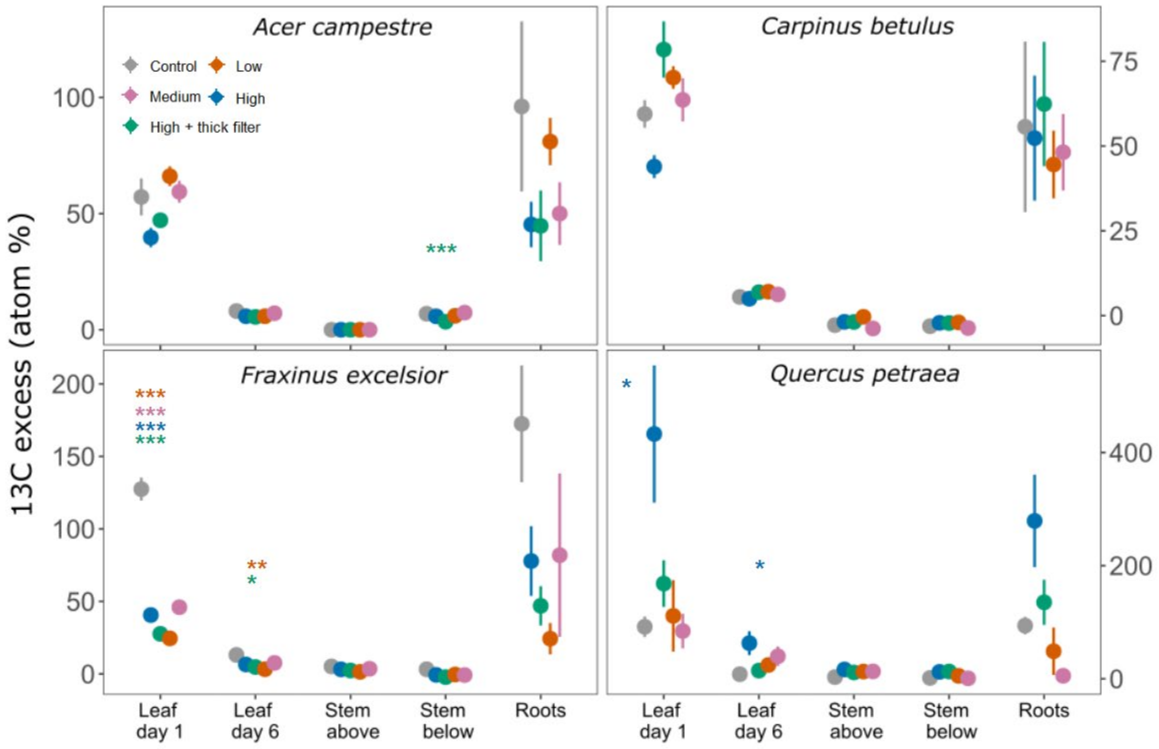

Supplementary Figure S3. Mean (±SE, n=4) carbon isotopic (^{13}C) excess in the different tissues (roots, leaf, stem above and below the scanned zone) measured six days after the labeling (plus leaves also measured right after the labeling). Stars denote significant differences from the control for a given sample (*<0.05, **<0.01, ***<0.001). Note the different scales for the different species.

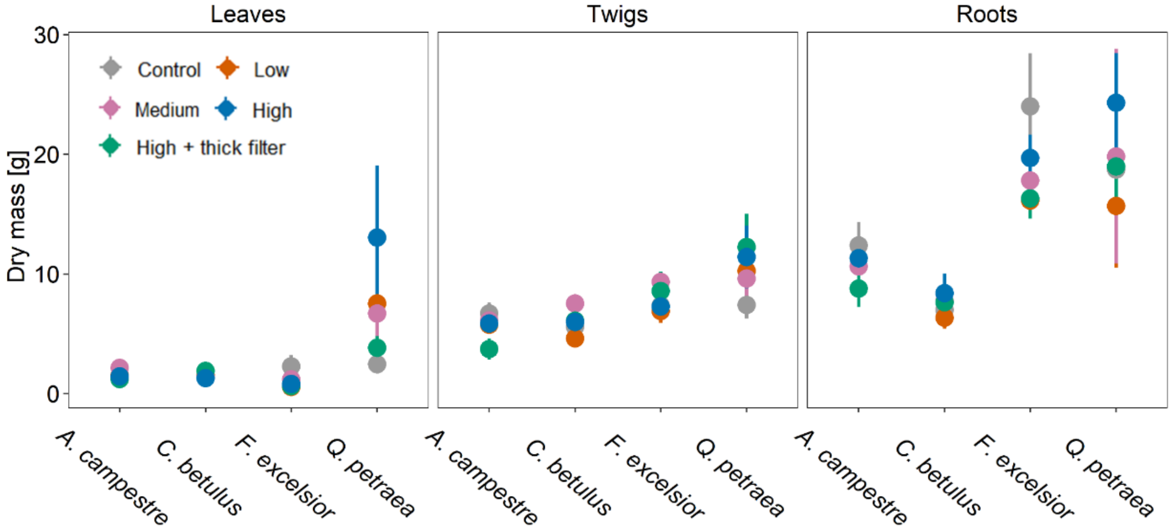

Supplementary Figure S4. Dry biomass (mean ± SE, n=4) of different tissues (leaves, roots, twigs including stems) sampled six days after the labeling. No significant differences were found between the control and the treatments.

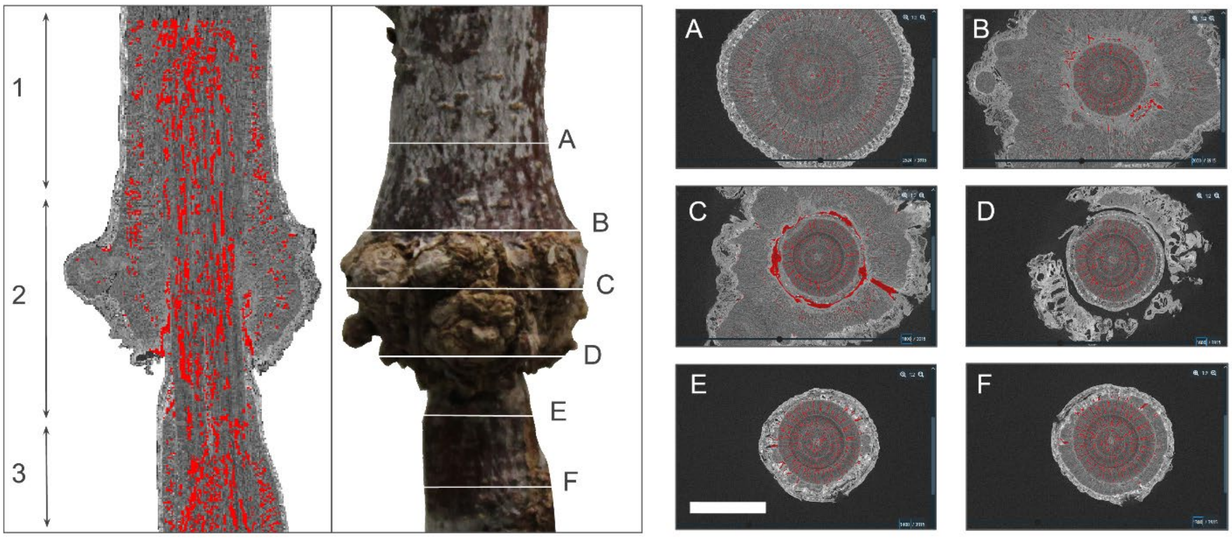

Supplementary Figure S5. Images from a 3D reconstruction (using Avizo 2021 software) of a 5-year-old European hornbeam tree stem deformed by X-ray radiation (resulting from µ-CT scan). White scale bar represents 5mm and is applicable to all six images on the right. The tree was scanned in 2021 (two years before the pictures were taken), at the PIXE platform at EPFL in Lausanne. The parameters used correspond to the high-energy treatment in the present study. The deformed stem was sampled and air-dried before being scanned again for observation in 2023. Red color represents volumes filled with air (i.e., xylem vessels - as the sample was dry - and interstices in the deformed bark). Pictures A-F show transversal views of the stem at the corresponding z-position on the left picture. Zones 1, 2 and 3 on the left delimit different radial growths: in zone 2 (approximately the zone that was µ-CT scanned), cross-sections show three annual growth rings (pictures B - C - D - E); in zone 3 (below the scanned area) the stem shows four annual growth rings (picture F); and finally in zone 1 (above the scanned area) the stem shows five annual growth rings (picture A).

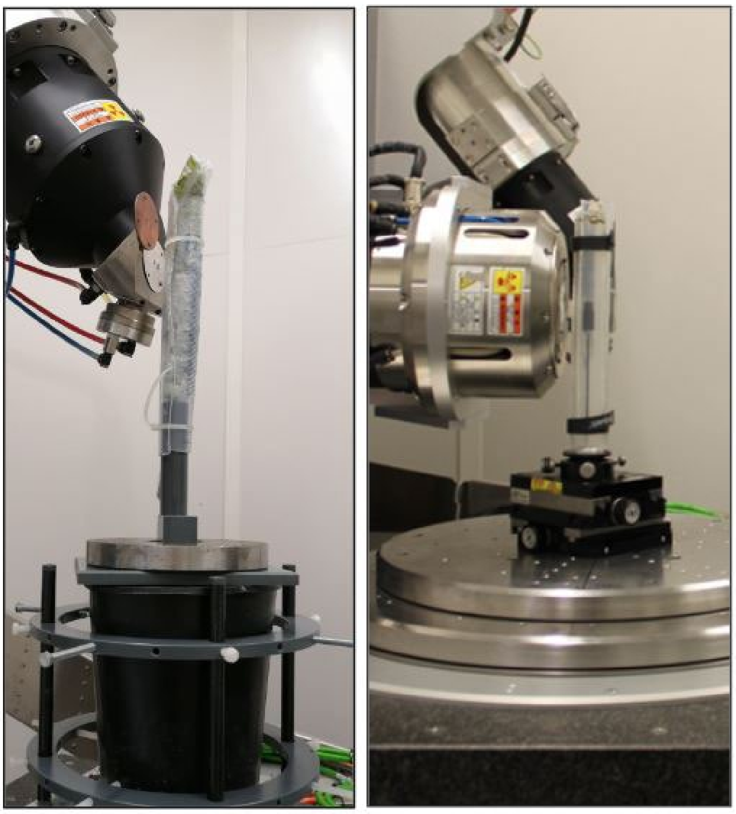

Supplementary Figure S6. Set-up at the PIXE µCT platform (EPFL, Lausanne, Switzerland) for *in-vivo* scanning of trees (left) and X-ray dose measurements (right). Trees/dosimeters were wrapped into plastic foil and held in a plastic cylinder to ensure stability during the measurements.

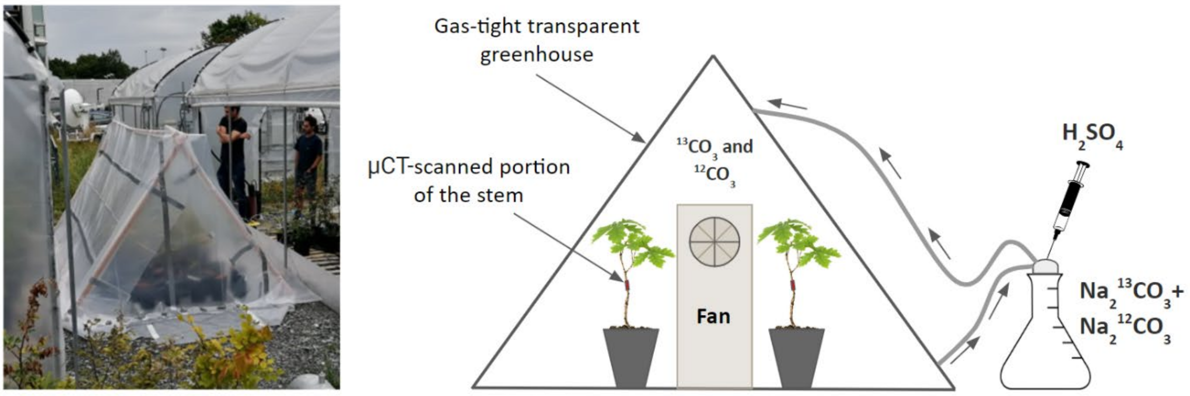

Supplementary Figure S7. Experimental set-up used for $^{13}CO_2$ labeling consisting of a sealed greenhouse with $^{13}CO_2$ injection. The gas-tight low-density polyethylene labeling greenhouse (4 × 2 × 1.5 m) used in the study permitted about 85% transmittance of photosynthetically active radiation. ^{13}C was applied as 50% $^{13}CO_2$ to the aboveground parts of the plants by pulse-labeling. One fan was installed in the center of the chamber to ensure the even distribution of $^{13}CO_2$. The $^{13}CO_2$ was released by mixing with H_2SO_4.
